# The Intellectual Disability Frailty Index Predicts 10‐Year Mortality Within the HA‐ID Cohort

**DOI:** 10.1111/jir.70111

**Published:** 2026-05-08

**Authors:** Marco C. van Maurik, Mylène N. Böhmer, Patrick J. E. Bindels, Dederieke A. M. Festen, Alyt Oppewal

**Affiliations:** ^1^ Department of General Practice, Intellectual Disability Medicine Research, Erasmus MC University Medical Center Rotterdam Rotterdam the Netherlands; ^2^ Academic Collaborative Research Center Healthy Ageing and Intellectual Disabilities Rotterdam the Netherlands; ^3^ Department of General Practice, Erasmus MC University Medical Center Rotterdam Rotterdam the Netherlands

**Keywords:** elderly, frail elderly, older adults, survival, vulnerable

## Abstract

**Background:**

Adults with intellectual disability (ID) experience frailty up to 20 years earlier than the general population, potentially increasing their risk of age‐related comorbidities and mortality at a younger age. This study investigates the relationship between frailty, assessed with the Intellectual Disability Frailty Index (ID‐FI) and its Short Form, and all‐cause mortality over 10 years in older adults with ID. Accurate mortality prediction may help identify high‐risk individuals and assist in creating targeted interventions for adults with ID.

**Methods:**

Data from 982 participants aged ≥ 50 years (mean age = 61.6 ± 8 years) with borderline to profound ID were analysed over a 10‐year follow‐up within the Healthy Ageing and Intellectual Disabilities (HA‐ID) cohort. Frailty was assessed using the 51‐item ID‐FI and the 17‐item ID‐FI Short Form, which measure frailty scores that can be classified into five categories: relatively fit, prefrail, mildly frail, moderately frail and severely frail. Cox proportional hazards models were used to assess the predictive validity of both indices for all‐cause mortality.

**Results:**

Of 982 study participants, 433 (44.1%) were deceased during 10‐year follow‐up. Higher frailty scores were significantly associated with increased mortality risk, independent of age, sex, level of ID and Down syndrome. Compared to relatively fit participants, the hazard ratios (HRs) for mortality for the ID‐FI were as follows: 1.53 (95% CI = 1.14–2.05) for prefrail, 3.17 (95% CI = 2.31–4.36) for mildly frail, 5.37 (95% CI = 3.66–7.89) for moderately frail and 10.00 (95% CI = 6.49–15.43) for severely frail participants. A similar pattern was demonstrated for the ID‐FI Short Form. Both indices demonstrated fair predictive accuracy (AUC = 0.72) for 10‐year all‐cause mortality.

**Conclusions:**

Both the ID‐FI and ID‐FI Short Form are predictive for 10‐year mortality risk in adults with ID. Future research should investigate how frailty changes over time and develop strategies to improve care for adults with ID.

## 1 Introduction

Frailty is a multifactorial biological syndrome related to ageing, characterised by a decline in physiological reserves across multiple organ systems, leading to increased vulnerability to stressors and a higher risk of adverse health outcomes and greater mortality risk (Dent et al. 2019). In the general population, frailty predicts higher care needs, falls, fractures, hospitalisations, lower quality of life and increased mortality risk (Clegg et al. 2013; Hoogendijk et al. 2014; Junius‐Walker et al. 2018; Vermeiren et al. 2016). Frailty is not a fixed state; it can increase over time or reverse (Dent et al. 2019; Fried et al. 2001; Junius‐Walker et al. 2018). The reversibility of frailty has led to a growing focus on frailty prevention and interventions to reduce frailty‐related risks in elderly care (Travers et al. 2023). Despite extensive research on the long‐term frailty‐related risks in the general elderly population (Dent et al. 2019; Jang et al. 2023), the long‐term risks of frailty on increased morbidity and mortality in older adults with intellectual disability (ID) remain largely unknown.

Increasing our understanding of frailty in older adults with ID is important, because this group tends to show signs of frailty much earlier (Schoufour et al. 2013). In fact, older adults with ID have frailty levels similar to those of adults in the general population who are 20 years older (Schoufour et al. 2013). This early onset of frailty could lead to serious consequences, such as a higher mortality risk at a younger age. Therefore, it is crucial to understand the long‐term frailty risks in older adults with ID, to help predict and reduce frailty‐related comorbidities and mortality. Increasing our knowledge of frailty will play an important role in the development of evidence‐based interventions aimed at improving healthy ageing in this population, for which new interventions are still necessary (El Mrayyan et al. 2025).

Adults with ID often face a range of health issues, including higher rates of motor and sensory disabilities, chronic conditions like epilepsy, mental health problems and dependency in performing activities of daily living (Schoufour et al. 2013, 2022). However, traditional frailty tools, designed for the general population, tend to overlook these specific challenges (Schoufour et al. 2022). This gap in measurement tools highlights the need for a frailty index that is specifically tailored to adults with ID, addressing the unique health concerns of this population.

To fill this gap, several frailty indices have been developed tailored to the ID population, such as the Home Care–Intellectual and Developmental Disabilities Frailty Index (HC‐IDD) and the Intellectual Disability Frailty Index (ID‐FI) (Martin et al. 2018; Schoufour et al. 2013). The ID‐FI and its short form were developed using data from the Healthy Ageing and Intellectual Disabilities (HA‐ID) cohort study (Schoufour et al. 2013, 2022) and are currently the most studied frailty index tailored to the ID population. While the ID‐FI has been shown to predict 3‐year all‐cause mortality in adults with ID (Schoufour et al. 2015), its ability to predict longer‐term outcomes, such as 10‐year mortality risk, remains unknown. Similarly, the ID‐FI Short Form showed promise in predicting 5‐year mortality (Schoufour et al. 2022). Comparably, the HC‐IDD has been shown to predict worsening frailty and mortality during a 12‐month follow‐up period, but long‐term mortality predictions have not yet been studied (Martin et al. 2018). As care transitions and interventions in adults with ID often require substantial time for planning and implementation, identifying 10‐year mortality risk may help support timely, proactive care planning.

This study aims to investigate the relationship between frailty in older adults with ID, as measured by the ID‐FI and its short form, and all‐cause mortality over a 10‐year follow‐up period. If the ID‐FI is a valid predictor of long‐term mortality risk, it can enable early identification of high‐risk individuals and support the development of targeted care strategies for adults with ID.

## 2 Methods

### 2.1 Study Design and Participants

This study is part of the longitudinal multicentre Healthy Ageing and Intellectual Disabilities (HA‐ID) cohort study, investigating health and ageing in older adults with ID. It is conducted within the Academic Collaborative Research Center Healthy Ageing and Intellectual Disabilities, a collaboration between the Department of General Practice, Intellectual Disability Medicine Research, Erasmus MC, University Medical Center Rotterdam, Rotterdam, the Netherlands, and three Dutch ID care organisations (Hilgenkamp et al. 2011).

The care organisations provide a wide range of care and support to individuals with ID, such as supported living, residential care and day activity programs. These organisations together covered about 10% of the Dutch ID population receiving formal care, making the base population representative for older adults with ID in the Netherlands receiving long‐term care and/or support (Hilgenkamp et al. 2011). All eligible individuals aged 50 years and over 61.6 ± 8 years within the care organisations were invited to participate; 1050 of 2322 (45.2%) eligible individuals took part in the baseline measurements. The resulting sample was largely representative of the target population, with a slight underrepresentation of those aged 80–84 years and more independent individuals, and a slight overrepresentation of women (de Leeuw et al. 2022; Hilgenkamp et al. 2011). Written informed consent was obtained from all participants or their legal representatives. Baseline data was collected between February 2009 and July 2010. The 10‐year follow‐up data collection began in October 2020 and was finished in April 2023 (de Leeuw et al. 2022). Due to delays in measurements caused by COVID‐19 lockdowns, the follow‐up period for some participants exceeded 10 years. Mean follow‐up time was calculated as the mean follow‐up time from baseline to death or censoring and was 8.8 years (SD 3.9; range = 0.1–14.7). Median follow‐up time, estimated using the reverse Kaplan–Meier method, was 11.6 years (95% confidence interval: 11.4–11.8). All measurements were completed by HA‐ID researchers, with the support of health care professionals from participating care organisations, among which physicians, behavioural therapists, speech therapists, physical therapists and nurses.

### 2.2 Ethical Approval

The Medical Ethics Review Committee of the Erasmus MC, University Medical Center Rotterdam, approved the study (MEC‐2008‐234, MEC‐2011‐309 and MEC‐2019‐0562), and we adhered to the guidelines of the Declaration of Helsinki (World Medical Association 2025).

### 2.3 Frailty

The ID‐FI (Schoufour et al. 2013) and the ID‐FI Short Form (Schoufour et al. 2022) were used to assess frailty. The ID‐FI and its short form consist of 51 and 17 items respectively. Participants were included if at least 30 of the 51 ID‐FI items could be scored, following Searle et al.'s guidelines for sufficiently accurate adverse event prediction using frailty indices (Rockwood et al. 2006; Searle et al. 2008). Both the ID‐FI and ID‐FI Short Form provide a frailty score between 0 and 1, calculated by dividing the total index score by the number of successfully completed items. A higher frailty score indicates a higher level of frailty (Schoufour et al. 2013). The items in the frailty indices cover a wide range of themes (Schoufour et al. 2013, 2022), including basic daily tasks (e.g., grocery shopping and dressing), somatic health indicators (e.g., bladder control and heart disease), psychological factors (e.g., listlessness and depression), social engagement (e.g., attending day‐care and social interactions) and laboratory measurements (e.g., cholesterol and glucose levels). The data used to calculate the indices were sourced from baseline measurements performed within the HA‐ID cohort study. A summary of the performed measurements that together form the ID‐FI can be found in Supplementary Table S1.

The ID‐FI Short Form was derived from the full ID‐FI based on statistical analysis, clinical and practical feasibility and input from an expert panel, aiming for a more practical assessment tool for frailty in adults with ID. Its validity was established by an acceptable internal consistency, strong correlation with the original index, alignment in frailty categorisation and a demonstrated association with (5 year) survival outcomes (Schoufour et al. 2022).

Frailty scores are categorised as relatively fit (ID‐FI score < 0.20), prefrail (ID‐FI score 0.20–0.29), mildly frail (ID‐FI score 0.30–0.39), moderately frail (ID‐FI score 0.40–0.49) and severely frail (ID‐FI score ≥ 0.50) for both indices (Schoufour et al. 2013). More information on the source of these measurements is published elsewhere (Hilgenkamp et al. 2011; Schoufour et al. 2013, 2022).

### 2.4 Participant Characteristics

Participant characteristics were collected at baseline. Information on age, sex and living circumstances (central setting, community based, independent with ambulatory support, with relatives and unknown) was collected from the administrative systems of the care organisations. Information on the level of ID (borderline, mild, moderate, severe or profound ID) was collected from the behavioural expert files. Information on the presence of Down syndrome (yes, no or unknown) and multimorbidity (having four or more chronic conditions) was collected from medical files (Hermans and Evenhuis 2014).

Professional caregivers provided information on the ability to perform basic and instrumental activities of daily living (ADL and IADL) by filling out the Barthel Index and the Lawton IADL scale (Lawton and Brody 1969; Mahoney and Barthel 1965). Both measurements have shown suitability for individuals with ID in the HA‐ID cohort (Schoufour et al. 2014). The Barthel Index measures basic self‐care tasks (ADL) across 10 items, such as bathing, dressing and eating, with scores ranging from 0 (completely dependent) to 20 (completely independent) (Mahoney and Barthel 1965). Its validity, test–retest reliability, sensitivity and clinical utility are good in the general population (Green et al. 2001; Gresham et al. 1980; Wade and Collin 1988; Wade and Hewer 1987).

The Lawton IADL scale, reported by professional caregivers, assesses complex skills required for independent living across eight items, such as managing finances, medication and transportation, with scores ranging from 8 (completely dependent) to 24 (completely independent). It has been used in hospitalised older patients (Albert et al. 2006), with a good correlation between self‐rated scores by older adults with dementia and ratings by clinicians (Albert et al. 2006).

### 2.5 All‐Cause Mortality

All‐cause mortality data was collected between February 2009 and April 2023. Information on the status of participants (survivors, deceased, lost to follow up or status unknown) was obtained from the administration systems of the care organisations. If participants were deceased, the date of death was provided. Survivors were censored at their last researcher contact date. If participants were lost to follow up due to relocation, the date of relocation was used as the censor date. If the date of relocation was not available, the last known contact date with the researchers was used as the censor date. If the status of participants could not be retrieved and was therefore unknown, these participants were excluded from analysis.

### 2.6 Statistical Analyses

Descriptive statistics were used to present baseline characteristics for the total study group. Chi‐square (χ^2^) tests (categorical data) and independent *t*‐tests (continuous data) were used to assess differences in baseline characteristics between deceased participants, survivors, those lost to follow‐up and those with status unknown.

Four Cox proportional hazards models were used to examine frailty's independent effect on mortality, using both continuous frailty scores and frailty categories from the ID‐FI and ID‐FI Short Form as dependent variables. Each model was adjusted for age, sex, level of ID and Down syndrome, with hazard ratios (HRs) and 95% confidence intervals (CIs) calculated. For interpretation clarity, ID‐FI scores were multiplied by 100 in the models.

The proportionality assumption for each Cox model was tested using Schoenfeld residuals. Kaplan–Meier estimates were used to generate survival curves across frailty categories for both the ID‐FI and its short form.

To assess the predictive accuracy of the ID‐FI and its short form for mortality, receiver operating characteristic (ROC) curves were generated, with the area under the curve (AUC) quantifying overall predictive accuracy (0.90–1.00 excellent, 0.80–0.90 good, 0.70–0.80 fair, 0.60–0.70 poor and 0.50–0.60 no discrimination).

All statistical analyses were conducted using SPSS Version 28.0.1.0 (142) and the survival package (Version 3.8–3) in R Version 4.3.2 (RStudio interface).

## 3 Results

Of the 1050 baseline participants, 982 participants had data available to complete at least 30 items on the ID‐FI. For all 982 participants, all items on the ID‐FI Short Form were available. The mean age at baseline was 62 years (SD = 8, range = 50–93), and 51.6% were male. At the 10‐year follow‐up, there were 469 survivors (47.8%), 433 (44.1%) were deceased, 38 (3.9%) were lost to follow‐up because of relocation, and the status of the remaining 42 (4.3%) was unknown.

Participants (*n* = 940) had higher frailty scores (*p* < 0.001), were less independent in daily functioning (ADL *p* < 0.001; IADL *p* < 0.001) and lived more often in a central setting (*p* < 0.001) than those with status unknown. There were no differences between participants and those with unknown status regarding age (*p* = 0.07), presence of Down syndrome (*p* = 0.4) or degree of intellectual disability (p = 0.07). Participants with status unknown were excluded from further analysis.

The mean time to mortality was 6.2 ± 3.7 years (range = 0.1–13.9 years). Deceased participants were on average older at baseline (*p* < 0.001), lived more often in a central setting (*p* < 0.001), more often had Down syndrome (*p* < 0.001), were less independent in daily functioning (ADL *p* < 0.001; IADL *p* < 0.001) and had higher frailty scores (*p* < 0.001) than survivors (Table 1). Of the deceased participants (*n* = 433), 78 participants (18.0%) were in the relatively fit group at baseline, 104 participants (24.0%) in the prefrail group, 107 participants (24.7%) in the mildly frail group, 94 participants (21.7%) in the moderately frail group, and 50 participants (11.5%) were in the severely frail group. Participant characteristics for each frailty category at baseline have been published previously; please refer to Supplementary Table 2 (S2) for more information (Schoufour et al. 2015).

**TABLE 1:** Baseline characteristics of the survivors, deceased, lost to follow‐up or status unknown.

Characteristic	Total group (*N* = 940)	Survivors (*n* = 469)	Deceased (*n* = 433)	Lost to follow‐up (*n* = 38)	Unknown (*n =* 42)
**Age**, mean ± SD	61.7 ± 8.1	59.4 ± 6.5 ^a^	64.5 ± 9.0	59.1 ± 5.5	59.4 ± 6.8
**Sex**, *n* (%)					
Male	484 (51.5)	237 (50.5)	232 (53.6)	15 (39.5)	23 (54.8)
Female	456 (48.5)	232 (49.5)	201 (46.4)	23 (60.5)	19 (45.2)
**Level of ID**, *n* (%)					
Borderline	28 (2.9)	20 (4.3)	7 (1.6)	1 (2.6)	2 (4.7)
Mild	195 (20.7)	100 (21.3)	88 (20.3)	7 (18.4)	12 (28.5)
Moderate	447 (47.5)	218 (46.5)	210 (48.5)	19 (50.0)	23 (54.7)
Severe	161 (17.1)	82 (17.5)	73 (16.9)	6 (15.7)	3 (7.1)
Profound	89 (9.4)	39 (8.3)	46 (10.6)	4 (10.5)	0
Unknown	20 (2.1)	10 (2.1)	9 (2.1)	1 (2.6)	2 (4.7)
**Down syndrome**, *n* (%)					
Yes	139 (14.7)	30 (6.4) ^a^	101 (23.3)	8 (21.0)	3 (7.1)
No	706 (75.1)	384 (81.9)	300 (69.3)	22 (57.8)	26 (61.9)
Unknown	95 (10.1)	55 (11.7)	32 (7.4)	8 (21.0)	13 (30.9)
**Living circumstances**, *n* (%)					
Central setting	534 (56.8)	233 (49.7)	291 (67.2)	10 (26.3)	0 ^b^
Community	363 (38.6)	212 (45.2)	128 (29.6)	23 (60.5)	32 (76.1)
Independent with ambulatory support	34 (3.6)	21 (4.5)	12 (2.8)	1 (2.5)	5 (11.9)
With relatives	3 (0.3)	0	1 (0.2)	2 (5.2)	2 (4.7)
Unknown	6 (0.6)	3 (0.6)	1 (0.2)	2 (5.2)	3 (7.1)
**ADL** (range = 0–20), mean ± SD	13.7 ± 5.8	15.3 ± 4.9 ^a^	11.9 ± 6.3	13.9 ± 5.3	17.1 ± 2.9 ^b^
**IADL** (range = 8–24), mean ± SD	11.7 ± 4.6	12.6 ± 5.1 ^a^	10.7 ± 4.0	11.4 ± 4.0	14.4 ± 4.8 ^b^
**ID‐FI frailty score**, mean ± SD	0.28 ± 0.1	0.23 ± 0.1 ^a^	0.33 ± 0.1	0.26 ± 0.1	0.19 ± 0.1 ^b^

Abbreviations: ID = intellectual disability; ID‐FI = Intellectual Disability Frailty Index; SD = standard deviation.

^a^ Indicating a significant difference between survivors and deceased participants (*p* < 0.001).

^b^ Indicating a significant difference between participants in the total study group and the unknown group (*p* < 0.001).

### 3.1 Survival Analysis

The ID‐FI continuous score was significantly related to mortality after adjusting for age, sex, presence of Down syndrome and level of ID, with an HR of 1.06 (95% CI = 1.05–1.07), representing a 6% increase in mortality risk for each 0.01‐point increase in ID‐FI score (Table 2). In comparison, the HR for age was also 1.06 (95% CI = 1.04–1.07). The frailty categories based on the ID‐FI score were also significantly related to mortality, with increasing HRs corresponding to increasing severity of frailty. Compared to relatively fit participants, prefrail participants had an HR of 1.53 (95% CI = 1.14–2.05), mildly frail participants had a mortality risk (HR) of 3.17 (95% CI = 2.31–4.36, *p* < 0.001), moderately frail participants had a mortality risk (HR) of 5.37 (95% CI = 3.66–7.89, *p* < 0.001) and severely frail participants had a mortality risk (HR) of 10.00 (95% CI = 6.49–15.43, *p* < 0.001) (Table 2).

**TABLE 2:** Cox proportional hazards models for predicting mortality with the ID‐FI and ID‐FI Short Form (*n* = 920).

ID‐FI with continuous frailty score				
Covariate	HR	95% CI (HR)	Wald Statistic	*p*
Continuous frailty score	1.06	(1.05, 1.07)	185.9	< 0.001
Age (per year)	1.06	(1.04, 1.07)	63.5	< 0.001

ID‐FI Short Form with continuous frailty score				
Covariate	HR	95% CI (HR)	Wald Statistic	*p*
Continuous frailty score	1.05	(1.04, 1.05)	172.8	< 0.001
Age (per year)	1.06	(1.04, 1.07)	68.3	< 0.001

ID‐FI with frailty categories				
Covariate	HR	95% CI (HR)	Wald Statistic	*p*
Prefrail	1.53	(1.14, 2.05)	8.0	< 0.01
Mildly frail	3.17	(2.31, 4.36)	50.6	< 0.001
Moderately frail	5.37	(3.66, 7.89)	73.5	< 0.001
Severely frail	10.00	(6.49, 15.43)	108.5	< 0.001
Age (per year)	1.06	(1.04, 1.07)	60.17	< 0.001

ID‐FI Short Form with frailty categories				
Covariate	HR	95% CI (HR)	Wald Statistic	*p*
Prefrail	1.50	(1.10, 2.03)	6.8	< 0.01
Mildly frail	2.73	(196, 3.81)	34.8	< 0.001
Moderately frail	3.51	(2.40, 5.13)	42.1	< 0.001
Severely frail	7.84	(5.36, 11.4)	113.8	< 0.001
Age (per year)	1.06	(1.10, 2.03)	78.4	< 0.001

*Note:* This table presents Cox proportional hazards models showing hazard ratios (HRs) with 95% confidence intervals for each covariate. Out of 980 eligible participants, 62 were excluded from these analyses because either their level of intellectual disability or they were in the unknown (21) group. ‘Relatively fit’ (frailty category) and ‘borderline and mild’ (intellectual disability group) served as reference groups in the Cox models and were therefore not included in the model outputs. All models were corrected for age, sex, the presence of Down syndrome and the level of intellectual disability.

Abbreviations: CI = confidence interval, HR = hazard ratio, ID = intellectual disability, ID‐FI = Intellectual Disability Frailty Index.

The ID‐FI Short Form continuous score was significantly related to mortality, with an HR of 1.05 (95% CI = 1.04–1.05; *p* < 0.001), representing a 5% increase in mortality risk for each 0.01‐point increase in ID‐FI Short Form score (Table 2). In comparison, the HR for age was also 1.06 (95% CI = 1.04–1.07; *p* < 0.001). The frailty categories based on the ID‐FI Short Form were also significantly related to mortality, with increasing HRs corresponding to increasing severity of frailty (Table 2). Compared to relatively fit participants, prefrail participants in the ID‐FI Short Form had an HR of 1.50 (95% CI = 1.10–2.03, *p* < 0.01), mildly frail participants an HR of 2.73 (95% CI = 1.96–3.81, *p* < 0.001), moderately frail participants had a mortality risk of 3.51 (HR) (95% CI = 2.40–5.13, *p* < 0.001), and severely frail participants had a mortality risk of 7.84 (HR) (95% CI = 5.37–11.44, *p* < 0.001).

**FIGURE 1:**
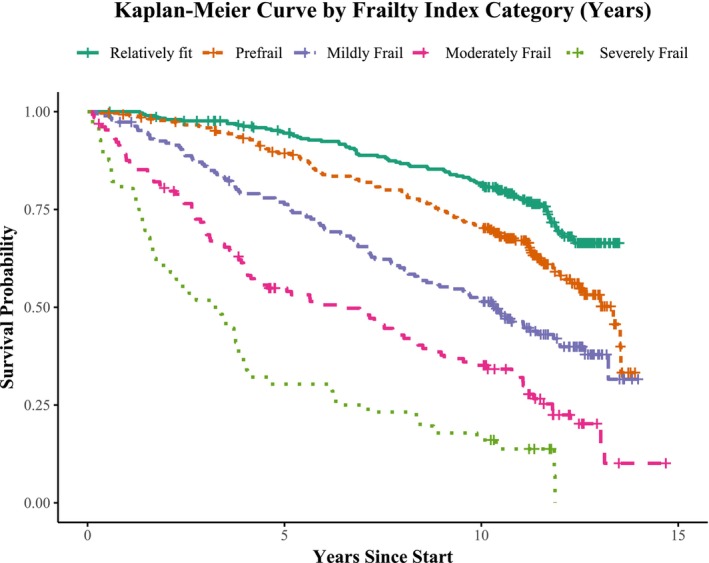
Fifteen‐year survival by frailty category measured with the Intellectual Disability Frailty Index.

**FIGURE 2:**
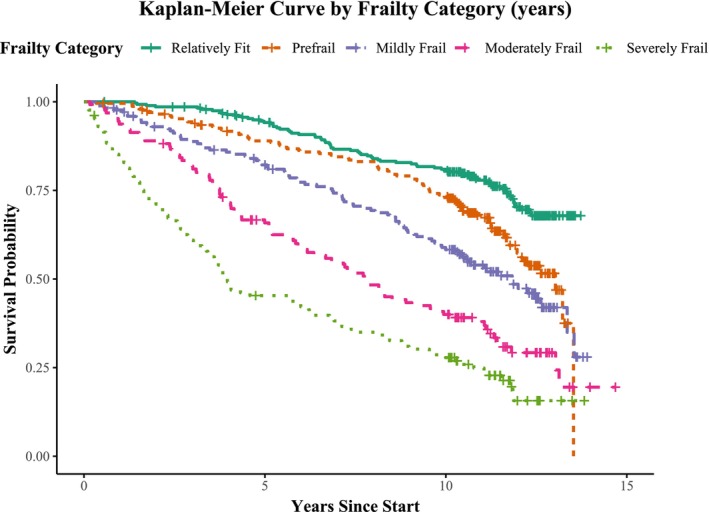
Fifteen‐year survival by frailty category measured with the Intellectual Disability Frailty Index Short Form.

Kaplan–Meier curves were created for frailty categories of the ID‐FI (Figure 1) and the ID‐FI Short Form (Figure 2). Both Kaplan–Meier curves visualise the significant difference in survival probabilities across frailty categories (*p* < 0.001). At 5 years, survival in the relatively fit group was 95% (ID‐FI) and 94% (ID‐FI Short Form), compared to 30% (ID‐FI) and 45% (ID‐FI Short Form) in the severely frail group. At 10 years, survival was 82% (ID‐FI) and 81% (ID‐FI Short Form) in the relatively fit group, and 16% (ID‐FI) and 28% (ID‐FI Short Form) in the severely frail group. A consistent decline in survival was observed across increasing frailty scores for both indices.

**FIGURE 3:**
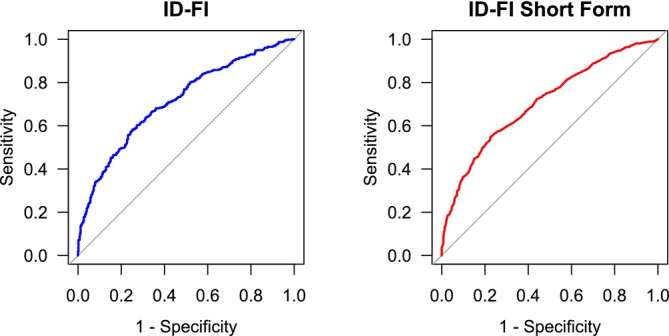
ROC curves for predicting 10‐year mortality for the ID‐FI and ID‐FI Short Form. Receiver Operating Characteristic (ROC) Curves for ID‐FI (left) and ID‐FI Short Form (right). Each curve illustrates the trade‐off between sensitivity and specificity providing insights into the accuracy and utility of each index in predicting mortality events among older adults with ID. ID‐FI = Intellectual Disability Frailty Index.

The ROC analyses to assess the predictive accuracy of the ID‐FI for mortality showed an AUC of 0.72 (95% CI = 0.69–0.75, *p* < 0.001), indicating a fair discriminative ability between those at risk for mortality and those not at risk (*p* < 0.001; Figure 3).

## 4 Discussion

This study investigated the relationship between frailty, as measured by the ID‐FI and ID‐FI Short Form, and all‐cause mortality over a 10‐year follow‐up period in older adults with ID. Both indices were found to be fair predictors for long‐term mortality, independent of age, sex, level of ID and presence of Down syndrome. Each unit increase in frailty score, whether measured by the ID‐FI (HR = 1.06; 95% CI = 1.05–1.07; *p* < 0.001) or the ID‐FI Short Form (HR = 1.05; 95% CI = 1.04–1.05; *p* < 0.001), was associated with a higher risk of mortality of 5%–6%. Even after adjusting for age, sex, level of ID and the presence of Down syndrome, the ID‐FI and its short form retained their predictive accuracy, highlighting their potential as tools to identify individuals at high risk. The Kaplan–Meier curves show a steeper decline in survival for participants with higher frailty scores or categories compared to those who were relatively fit. The higher mortality risk for each frailty category reinforces the validity of these frailty categories.

The findings of this study align with earlier research on the predictive validity of the ID‐FI over a 3‐year follow‐up period, where an HR of 1.08 indicated an 8% increase in mortality risk for every 0.01‐point increase in the ID‐FI score (Schoufour et al. 2015). In comparison, the HR of 1.06 observed in this study suggests a slightly lower predictive value for mortality risk over a 10‐year follow‐up. This lower predictive value is expected because baseline frailty measurements were used to predict outcomes over a much longer period than 3 years. Nevertheless, a strong association persists between higher frailty scores and categories and increased mortality risk, even after adjusting for age.

When comparing our findings with another frailty index developed specifically for individuals with ID, the HC‐IDD, we see similarities (Martin et al. 2018). During a 12‐month follow‐up period (mean follow‐up = 8.3 ± 2.2 months) the HC‐IDD demonstrated that being prefrail at baseline was associated with an increased risk of frailty worsening or mortality (RR = 1.24, 95% CI = 1.04–1.49). These findings support that frailty indices developed for people with ID consistently show some degree of association with mortality. Our findings contribute to this growing body of evidence by demonstrating the predictive validity of the ID‐FI indices, reinforcing the importance of frailty assessment tools tailored to the ID population.

When compared to a widely used frailty index for the general population developed by Rockwood et al., the ID‐FI shows similar predictive validity for mortality. Rockwood et al. reported HRs between 1.03 and 1.05 per 0.01‐point increase over a period of up to 14 years in the general population (Kojima et al. 2018; Rockwood et al. 2011), which is comparable to our findings in the ID population. Similarly, in a Korean general population cohort, an HR of 1.06 per 0.01‐point increase in the frailty index was observed over a 10‐year follow‐up period (Jang et al. 2023). These findings indicate that the ID‐FI has predictive validity for mortality in the ID population comparable to that of frailty indices applied in the general population over a similar time period. As previous studies have shown that general population frailty indices may misclassify frailty in adults with ID (Festen et al. 2021), the present results provide strong support for the ID‐FI as a more suitable tool for frailty assessment in this population.

Both the full ID‐FI and its short form demonstrate comparable predictive validity for mortality based on the ROC curves. This indicates that the indices can be used interchangeably. Although both indices were developed through statistical analyses and validated with HA‐ID cohort data, both indices have yet to be tested in clinical practice. Consequently, it is essential to evaluate their clinical usability and interrater reliability as standalone tools. Usability studies should address the application of the frailty indices in a clinical setting, while reliability studies should focus on consistency among healthcare professionals, which is critical for screening and monitoring frailty progression. Although current results are promising, further research is vital to assess the usability and reliability of these tools in clinical practice.

Relatively fit participants showed resilience throughout the 10‐year follow‐up, with significantly lower mortality rates compared to those in higher frailty categories. This highlights the importance of understanding the factors that contribute to resilience and prolonged survival. Identifying what protects this group is critical for developing strategies to maintain health and delay the onset of frailty, particularly in populations at higher risk, such as older adults with ID. The transition from being relatively fit to becoming frail marks a turning point where mortality risk sharply increases. Without a clear understanding of these turning points, opportunities for early intervention may be missed.

This study has several strengths and limitations. To our knowledge, this is the first large‐scale investigation using ID‐specific frailty indices with complete long‐term data to examine the relationship between frailty, ageing and mortality patterns in the ID population. The ID‐FI and its short form were specifically developed for individuals with ID, making them more appropriate than general population frailty indices for this purpose. All data were collected from the HA‐ID cohort, a large and nearly representative sample of older adults with ID, followed over a 10‐year period (Hilgenkamp et al. 2011). However, the reliance on baseline frailty in the HA‐ID cohort limits insight into how frailty levels may have changed over time. It is unknown whether frailty increased gradually or spiked shortly before death, making it difficult to assess how these patterns influence mortality risk. Identifying key turning points in frailty progression is crucial for developing timely interventions that could prevent or delay adverse health outcomes. Additionally, we saw a larger number of participants lost to follow‐up among those with mild ID living in community‐based settings, which may have introduced a bias towards individuals receiving centralised, specialised care. These individuals, being more independent and requiring less care, are more likely to relocate due to the broader range of care options available to them in the Netherlands. The skew towards participants receiving centralised, specialised ID care may limit the generalisability of our findings to more independent adults with ID. However, the HA‐ID cohort includes a significant portion (22.4%) of adults with borderline to mild ID and a small group (4.0%) that lives independently. Moreover, we found no significant differences in mortality prediction across various levels of ID, suggesting that while the bias towards moderate to severe ID may have some influence, it does not substantially affect the overall conclusions of this study.

To our knowledge, this study is the first to explore the relationship between frailty and mortality in a large cohort of older adults with ID over a 10‐year period, supported by a robust near‐representative sample (Hilgenkamp et al. 2011). The ID‐FI and its short form have demonstrated fair predictive validity for mortality in the ID population, making them an excellent replacement for the existing frailty indices which were made for the general population. These indices show comparable predictive validity to general frailty indices while addressing the specific needs and traits of the ID population. Although further validation in clinical practice is needed, the ID‐FI and its short form hold considerable potential to complement existing care practices, guide resource allocation, assist policymaking and support targeted interventions, both for adults with ID who are at highest risk and for maintaining the resilience of those who are not.

## Funding

This work is supported by ZonMw grant numbers 57 000 003, 314 030 302 and 839 180 001. In addition to external funding, the 10‐year follow‐up of the HA‐ID study is funded by the three Dutch care organisations, Abrona, Amarant and Ipse de Bruggen, involved in the HA‐ID consortium and the department of General Practice of the Erasmus MC, University Medical Center Rotterdam, the Netherlands.

## Disclosure

This work was sponsored by the Department of General Practice of the Erasmus MC, University Medical Center in Rotterdam, the Netherlands. The 10‐year follow‐up of the HA‐ID study was sponsored by the three Dutch care organisations, Abrona, Amarant and Ipse de Bruggen, involved in the HA‐ID Academic Collaborative Research Center, as well as the Department of General Practice of the Erasmus MC. The three care organisations were not involved in the data processing analyses, interpretation or preparation of the manuscript.

## Conflicts of Interest

The authors declare no conflicts of interest.

## Supporting information


**Table S1:** Baseline measurements within the HA‐ID study that together form the ID‐FI.
**Table S2:** Previously reported baseline characteristics of the HA‐ID cohort study according to frailty status measured by the ID‐FI (Schoufour et al. 2015).

## Data Availability

The data that support the findings of this study are available from the corresponding author upon reasonable request.
