# Supplementary material for: The Intellectual Disability Frailty Index Predicts 10‐Year Mortality Within the HA‐ID Cohort
Source: J Intellect Disabil Res. 2026 May 8;70(7):725–33. doi: 10.1111/jir.70111 (PMC13238440; doi:10.1111/jir.70111)
Supplement: Supplementary file 1 — Table S1: Baseline measurements within the HA‐ID study that together form the ID‐FI. Table S2: Previously reported baseline characteristics of the HA‐ID cohort study according to frailty status measured by the ID‐FI (Schoufour et al. 2015). [file JIR-70-725-s001.docx]

**Supplementary Table S1. Baseline measurements within the HA-ID study that together form the ID-FI.**

| **Type** | **Outcome** | **Details** |
| --- | --- | --- |
| Physical assessment | Brachial blood pressure | Omron M7 (OMRON Healthcare, the Netherlands). |
|  | Ankle-Arm-Index | Omron M7 (OMRON Healthcare, the Netherlands) (arm). Boso classico and 8-MHz Doppler probe (Huntleigh MD II, United Kingdom) (ankle). Ankle-arm-index calculated: systolic blood pressure ankle divided by systolic blood pressure arm. |
| Venipuncture | Biochemical markers | HDL, Haemoglobin |
| Medical file | Cardiovascular disease | Presence of CVD (heart failure, myocardial infarction, stroke, transient ischemic attack (TIA), cardiac arrhythmias, angina pectoris, aortic aneurysm, peripheral arterial disease (PAD), hypertension etc.), CVD risk factors (diabetes mellitus, central obesity, metabolic syndrome, rheumatoid arthritis, incriminating family history etc.) and treatments/  interventions (revascularisation of the coronary artery, pacemaker and implantable cardioverter-defibrillator (ICD)). |
|  | Endocrine disorders | Presence of endocrine disorders (such as diabetes mellitus, hypercholesterolemia and metabolic syndrome). |
| Fitness assessment | Manual dexterity | Box and block test (Mathiowetz et al., 1985). |
|  | Balance | Comfortable and maximum walking speed (5m) (Oppewal and Hilgenkamp, 2020). |
|  | Grip strength | Jamar Hand Dynamometer (#5030J1, Sammons Preston Rolyan, USA)(Oppewal and Hilgenkamp, 2020). |
| Questionnaires professional caregiver | Mobility | Self-assembled questionnaire based on the Hauser Ambulation Index and the characteristics of the Gross Motor Function Classification Scale (Hauser et al., 1983; Palisano et al., 1997). |
| Medical file |  | Scoliosis |
| Questionnaires professional caregiver | Informant-report depression and anxiety | Anxiety, Depression and Mood Scale (ADAMS) (Esbensen et al., 2003). |
|  |  | Signaallijst Depressie Zwakzinnigen (SDZ) (Kraijer and Plas, 2006). |
|  | Cognitive functioning | Dementia questionnaire for people with intellectual disabilities (DMR) (Evenhuis, 1995). |
| Physical assessment | Body Mass Index | Calculated from height and weight |
|  | Body circumferences | Measuring tape for hip, calf and upper arm circumference. |
|  | Bone Quality | Ultrasonometer (Lunar Achilles Insight, GE Healthcare, United States) for measuring bone stiffness calcaneus. |
| Meal time observation | Dysphagia | Dysphagia Disorder Survey (Sheppard, 1991). |
| Questionnaires professional caregiver | Malnutrition | Mini Nutritional Assessment (MNA) (Vellas et al., 1999). |
|  |  | Short Nutritional Assessment Questionnaire for Residential Care (SNAQRC) (Kruizenga et al., 2010). |
|  | Eating disorders | Screening Tool of feeding Problems (STEP) (Matson and Kuhn, 2001). |
| Medical file | Gastrointestinal diseases | Presence of gastrointestinal disease in the medical file (such as gastroesophageal reflux disease, gastric ulcer, constipation and dysphagia). |
|  | Malignancies | Presence of malignancies in the medical file. |
|  | Pulmonary diseases | Presence of pulmonary disease in the medical file (such as asthma, chronic obstructive pulmonary disease and sleep apnoea syndrome). |
|  | Visual and hearing impairments | Presence of visual and hearing impairments in the medical file. |
|  | Medication use | Medication use (medicament and dosage) as stated in the medical file. |
| Questionnaires professional caregiver | Activities of daily life | Barthel Index (Mahoney and Barthel, 1965). |
|  | Instrumental activities of daily life | Questionnaire based on the Instrumental Activities of Daily Living of Lawton and Brody and the Groningen Activities Restriction Scale (Kempen et al., 1996; Lawton and Brody, 1969). |
|  | Daytime activities | Self-assembled questions about daytime activities and/or work of the participant. |

CVD, cardiovascular disease; HDL, high-density lipoprotein; ICD-10, International Classification of Diseases 10th revision

**Supplementary Table S2:** Previously reported Baseline Characteristics of the HA-ID Cohort Study According to Frailty Status measured by the ID-FI (Schoufour et al., 2015).

| **Characteristic** |  | **Relatively Fit (FI < 0.20)**  **n = 325** | **Vulnerable (FI 0.20–0.29)**  **n = 279** | **Mildly Frail (FI = 0.30–0.39)**  **n = 192** | **Moderately Frail (FI = 0.40–0.49)**  **n = 130** | **Severely Frail (FI ≥ 0.50)**  **n = 56** | ***p*-valueᵃ** |
| --- | --- | --- | --- | --- | --- | --- | --- |
| **Age, mean±SD** |  | 60±6.2 | 61±7.7 | 63±9.3 | 65±9.3 | 68±10.5 | < .001 |
| **Sex, n (%)** |  |  |  |  |  |  | .16 |
| Male |  | 176 (54.2) | 151 (54.1) | 83 (43.2) | 65 (50) | 31 (55.4) |  |
| Female |  | 149 (45.8) | 128 (45.9) | 109 (56.8) | 65 (50) | 25 (44.6) |  |
| **Level of ID, n (%)ᵇ** |  |  |  |  |  |  | <.001 |
| Borderline |  | 18 (5.5) | 7 (2.5) | 4 (2.1) | 0 (0.0) | 1 (1.8) |  |
| Mild |  | 115 (35.4) | 48 (17.2) | 24 (12.5) | 14 (10.8) | 6 (10.7) |  |
| Moderate |  | 167 (51.4) | 150 (53.8) | 84 (43.8) | 50 (38.5) | 19 (33.9) |  |
| Severe |  | 19 (5.8) | 54 (19.4) | 45 (23.4) | 34 (26.2) | 13 (23.2) |  |
| Profound |  | 1 (0.3) | 12 (4.3) | 28 (14.6) | 31 (23.8) | 17 (30.4) |  |
| **Down syndrome, n (%)ᶜ** |  |  |  |  |  |  | .03 |
| Yes |  | 32 (9.8) | 46 (16.5) | 30 (15.6) | 20 (15.4) | 14 (25.0) |  |
| No |  | 220 (67.7) | 195 (69.9) | 140 (72.9) | 90 (69.2) | 40 (71.4) |  |
| **ADL** (range 0-20), mean±*SD* |  | 18.4±1.9 | 15.5±2.9 | 11.6±4.3 | 6.68±4.7 | 2.84±3.47 | <.001 |
| **IADL** (range 8-24), mean±*SD* |  | 15.8±4.9 | 11.1±3.7 | 9.39±2.4 | 8.68±1.8 | 8.20±0.62 | <.001 |
| **Multimorbidity, n (%)^d^** |  | 55 (16.9) | 124 (44.4) | 137 (71.3) | 108 (83.1) | 55 (98.2) | <.001 |
| **Frailty index score, mean±SD** |  | 0.14±0.04 | 0.25±0.03 | 0.34±0.03 | 0.44±0.03 | 0.56±0.05 | <.001 |

This table was sourced from the paper by (Schoufour et al., 2015).

ᵃ Differences between the frailty groups assessed with the Spearman’s Rho.
ᵇ Data on level of intellectual disability (ID) were not available for 21 participants.
ᶜ Data on presence of Down syndrome were not available for 155 participants.
d Assessed using a list of 20 chronic conditions and defined as presence of ≥4 morbid conditions (Hermans and Evenhuis, 2014).

HA-ID = Healthy Ageing and Intellectual Disability

*ID-FI = Intellectual Disability Frailty Index*

*SD = Standard Deviation*

**Supplementary references**

de Leeuw, M.J., Oppewal, A., Elbers, R.G., Knulst, M.W.E.J., van Maurik, M.C., van Bruggen, M.C., Hilgenkamp, T.I.M., Bindels, P.J.E., Maes-Festen, D.A.M., 2022. Healthy Ageing and Intellectual Disability study: summary of findings and the protocol for the 10-year follow-up study. BMJ Open 12, e053499. https://doi.org/10.1136/bmjopen-2021-053499

Esbensen, A.J., Rojahn, J., Aman, M.G., Ruedrich, S., 2003. Reliability and validity of an assessment instrument for anxiety, depression, and mood among individuals with mental retardation. J Autism Dev Disord 33, 617–629. https://doi.org/10.1023/b:jadd.0000005999.27178.55

Evenhuis, H., 1995. Manual of the dementia questionnaire for persons with mental retardation (DMR).

Hauser, S.L., Dawson, D.M., Lehrich, J.R., Beal, M.F., Kevy, S.V., Propper, R.D., Mills, J.A., Weiner, H.L., 1983. Intensive Immunosuppression in Progressive Multiple Sclerosis. New England Journal of Medicine 308, 173–180. https://doi.org/10.1056/NEJM198301273080401

Hermans, H., Evenhuis, H.M., 2014. Multimorbidity in older adults with intellectual disabilities. Res Dev Disabil 35, 776–783. https://doi.org/10.1016/j.ridd.2014.01.022

Kempen, G.I.J.M., Miedema, I., Ormel, J., Molenaar, W., 1996. The assessment of disability with the Groningen Activity Restriction Scale. Conceptual framework and psychometric properties. Social Science & Medicine 43, 1601–1610. https://doi.org/10.1016/S0277-9536(96)00057-3

Kraijer, D., Plas, J., 2006. Handboek Psychodiagnostiek en beperkte begaafdheid: Classificatie, test- en schaalgebruik. Amsterdam.

Kruizenga, H.M., De Vet, H.C.W., Van Marissing, C.M.E., Stassen, E.E.P.M., Strijk, J.E., Van Bokhorst-De Van Der Schueren, M.A.E., Horman, J.C.H., Schols, J.M.G.A., Van Binsbergen, J.J., Eliens, A., Knol, D.L., Visser, M., 2010. The SNAQRC, an easy traffic light system as a first step in the recognition of undernutrition in residential care. The Journal of nutrition, health and aging 14, 83–89. https://doi.org/10.1007/s12603-009-0147-1

Lawton, M.P., Brody, E.M., 1969. Assessment of older people: self-maintaining and instrumental activities of daily living. Gerontologist 9, 179–186.

Mahoney, F.I., Barthel, D.W., 1965. FUNCTIONAL EVALUATION: THE BARTHEL INDEX. Md State Med J 14, 61–65.

Mathiowetz, V., Volland, G., Kashman, N., Weber, K., 1985. Adult Norms for the Box and Block Test of Manual Dexterity. Am J Occup Ther 39, 386–391. https://doi.org/10.5014/ajot.39.6.386

Matson, J.L., Kuhn, D.E., 2001. Identifying feeding problems in mentally retarded persons: development and reliability of the screening tool of feeding problems (STEP). Research in Developmental Disabilities 22, 165–172. https://doi.org/10.1016/S0891-4222(01)00065-8

Oppewal, A., Hilgenkamp, T.I.M., 2020. Adding meaning to physical fitness test results in individuals with intellectual disabilities. Disability and Rehabilitation 42, 1406–1413. https://doi.org/10.1080/09638288.2018.1527399

Palisano, R., Rosenbaum, P., Walter, S., Russell, D., Wood, E., Galuppi, B., 1997. Development and reliability of a system to classify gross motor function in children with cerebral palsy. Developmental Medicine & Child Neurology 39, 214–223. https://doi.org/10.1111/j.1469-8749.1997.tb07414.x

Schoufour, J.D., Mitnitski, A., Rockwood, K., Evenhuis, H.M., Echteld, M.A., 2015. Predicting 3-year survival in older people with intellectual disabilities using a Frailty Index. J Am Geriatr Soc 63, 531–536. https://doi.org/10.1111/jgs.13239

Sheppard, J.J., 1991. Managing dysphagia in mentally retarded adults. Dysphagia 6, 83–87. https://doi.org/10.1007/BF02493484

Vellas, B., Guigoz, Y., Garry, P.J., Nourhashemi, F., Bennahum, D., Lauque, S., Albarede, J.-L., 1999. The mini nutritional assessment (MNA) and its use in grading the nutritional state of elderly patients. Nutrition 15, 116–122. https://doi.org/10.1016/S0899-9007(98)00171-3
